# Development and evaluation of a usable blastocyst predictive model using the biomechanical properties of human oocytes

**DOI:** 10.1371/journal.pone.0299602

**Published:** 2024-05-02

**Authors:** Daniel Meyer, Jonathan Kort, Ching Hung Chen, Huan Zhao, Xiaoling Yi, Shin-Yu Lai, Farn Lu, Wen Jui Yang, I-Chiao Hsieh, Chung-Li Chiang, Wei-Ming Chen, Jack Yu Jen Huang, David Camarillo, Barry Behr

**Affiliations:** 1 Department of Bioengineering, Stanford University, Stanford, CA, United States of America; 2 Division of Reproductive Endocrinology and Infertility, Stanford University, Stanford, CA, United States of America; 3 Institute of Molecular and Cellular Biology, National Tsing Hua University, Hsinchu, Taiwan; 4 Department of Obstetrics and Gynecology, Ton Yen General Hospital, Hsinchu, Taiwan; 5 Taiwan IVF Group Center for Reproductive Medicine & Infertility, Hsinchu, Taiwan; 6 Department of Reproductive Medicine, The 3rd Affiliated Hospital of Shenzhen University, Shenzhen, China; 7 Department of Data Science, Inti Taiwan, Inc., Zhubei City, Hsinchu, Taiwan; University of Florida, UNITED STATES

## Abstract

**Purpose:**

The purposes of this study were to determine whether biomechanical properties of mature oocytes could predict usable blastocyst formation better than morphological information or maternal factors, and to demonstrate the safety of the aspiration measurement procedure used to determine the biomechanical properties of oocytes.

**Methods:**

A prospective split cohort study was conducted with patients from two IVF clinics who underwent in vitro fertilization. Each patient’s oocytes were randomly divided into a measurement group and a control group. The aspiration depth into a micropipette was measured, and the biomechanical properties were derived. Oocyte fertilization, day 3 morphology, and blastocyst development were observed and compared between measured and unmeasured cohorts. A predictive classifier was trained to predict usable blastocyst formation and compared to the predictions of four experienced embryologists.

**Results:**

68 patients and their corresponding 1252 oocytes were included in the study. In the safety analyses, there was no significant difference between the cohorts for fertilization, while the day 3 and 5 embryo development were not negatively affected. Four embryologists predicted usable blastocyst development based on oocyte morphology with an average accuracy of 44% while the predictive classifier achieved an accuracy of 71%. Retaining the variables necessary for normal fertilization, only data from successfully fertilized oocytes were used, resulting in a classifier an accuracy of 81%.

**Conclusions:**

To date, there is no standard guideline or technique to aid in the selection of oocytes that have a higher likelihood of developing into usable blastocysts, which are chosen for transfer or vitrification. This study provides a comprehensive workflow of extracting biomechanical properties and building a predictive classifier using these properties to predict mature oocytes’ developmental potential. The classifier has greater accuracy in predicting the formation of usable blastocysts than the predictions provided by morphological information or maternal factors. The measurement procedure did not negatively affect embryo culture outcomes. While further analysis is necessary, this study shows the potential of using biomechanical properties of oocytes to predict embryo developmental outcomes.

## Introduction

Since the 2012 committee opinion characterizing oocyte cryopreservation as non-experimental [1], patients undergoing fertility preservation, either for medical or planning purposes, are among the fastest growing patient populations pursuing assisted reproduction [2]. Guidance regarding how many oocytes to cryopreserve is usually provided based on extrapolations from large database studies on aneuploidy rates and maternal age [3] combined with anticipated attrition during fertilization, in vitro embryo development, and transfer success rates after euploid blastocyst transfer [4]. This data is not personalized and is largely based on an infertile population which may not be as applicable to a non-infertile patient population, since aneuploidy rates have been observed to vary by age [5]. The discovery of insufficient cryopreserved oocytes can be catastrophic for patients, as they may lose the opportunity to obtain viable ones. On the other hand, patients opting for potentially unnecessary additional oocyte preservation cycles to prevent unfavorable outcomes face associated costs and risks [6]. As such, improved assessments of individual oocyte’s reproductive potential are needed. Research in a variety of cell types has demonstrated that the biomechanical properties of cells reflect a cell’s intrinsic health or ability to function [7]. Often cellular function is dictated by its structure and biophysical properties [8]. Ovarian cancer cells have been shown to be softer than non-malignant epithelial cells, and softer cancer cells are more likely to metastasize than stiffer lower grade malignant cells [9]. In red blood cells, advancements in exploring RBC deformability changes induced by P. falciparum and understanding the biophysical parameters regulating their bio-rheological behavior have significantly improved in recent decades [10]. Given the critical changes in gamete and early embryo viscoelasticity, it is logical that alterations in the biomechanical properties of oocytes or embryos may offer insight into their fertilization, embryo viability, genetic abnormality, pregnancy [11–14]. To date, there is no standard guideline or technique to aid in the selection of oocytes that have higher likelihood of developing into usable blastocysts, which is chosen for transfer or vitrification. The purpose of this study was to demonstrate the safety of measuring the biomechanical (or viscoelastic) properties of oocytes using a similar micropipette aspiration device to the one used by Yanez et. al [15], while also determining whether biomechanical properties of oocytes correlate with preimplantation development in vitro.

## Methods

### Study design

**Fig 1:**
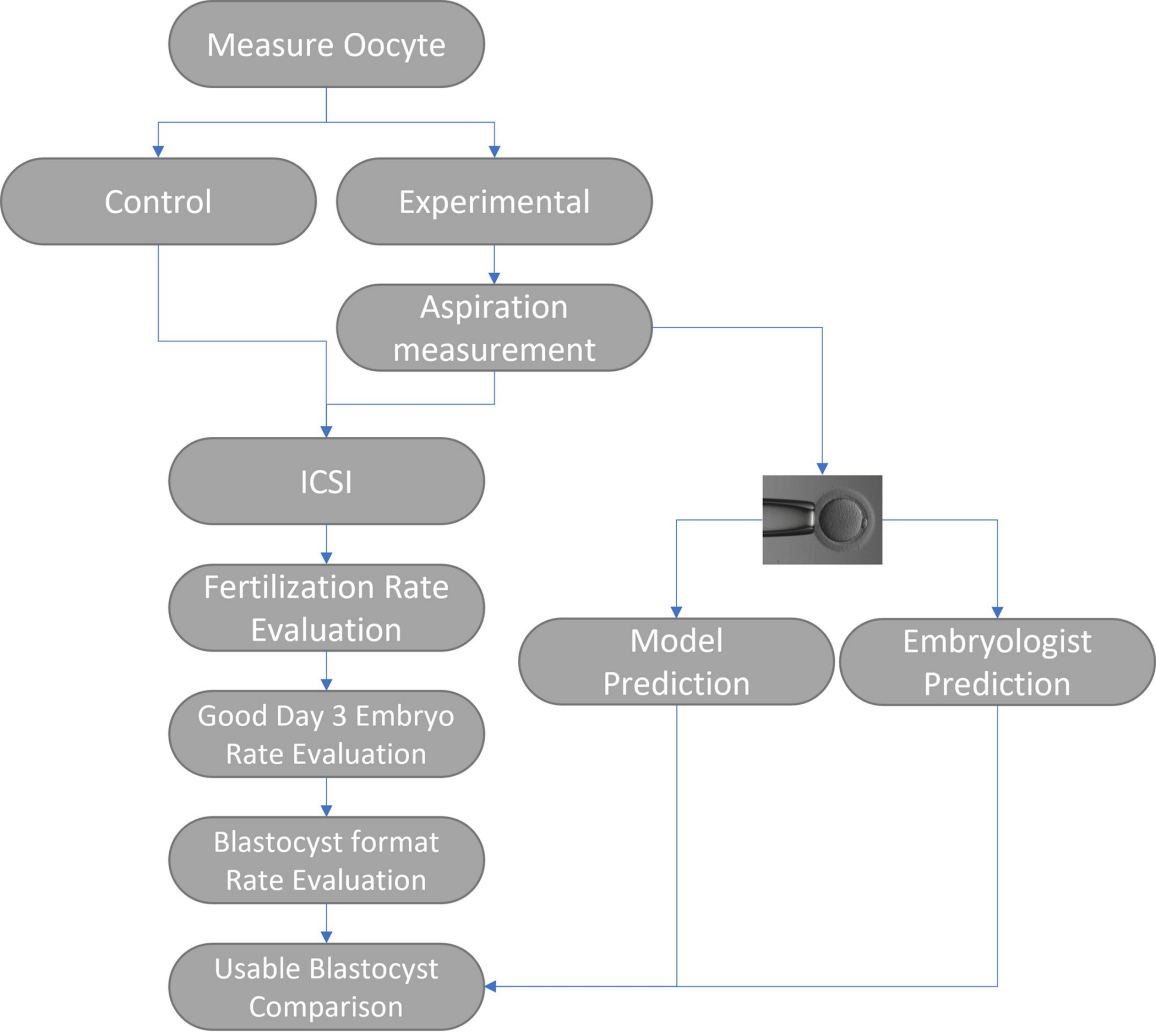
Workflow of study design. The oocytes were split into control and experimental groups to verify the safety of the oocyte after the aspiration measurement. Both groups followed the standard ICSI procedure to check the fertilization, good day 3 embryo, and blastocyst formation rate (see method of data analysis). Subsequently, the experimental group were further used to build a model to predict the usable blastocyst using biomechanical properties of oocytes (see method of determining the biomechanical properties of oocytes and data analysis). The accuracy of predicting usable blastocyst formation was compared between the model and the embryologists.

The cohort study (see Fig 1) from December 2016 to April 2021 (see Ethics Statement) was conducted to determine two tasks: 1) the safety of oocytes after the aspiration measurement procedure, and 2) the ability to select oocytes with a higher likelihood of developing into usable blastocysts using information on their biomechanical properties. To ensure an ample number of oocytes for analysis, specific inclusion criteria were established for patient recruitment: 1) the presence of more than 10 mature oocytes, 2) the development of more than 1 blastocyst, and 3) the age of women falling within the range of 21–45. The study population consisted of 46 patients recruited at the Shenzhen Army Hospital, along with their corresponding 279 measured and 480 unmeasured oocytes. On average, the patients were 29.85 years old (ranging from 21 to 39) and 16.74 MII oocytes were retrieved. At the Taiwan IVF Group Center, 22 patients were recruited. 215 oocytes were measured and 278 were used as control group. The average age of the patients was 34.36 years old (ranging from 28 to 41) and 22.45 MII oocytes were retrieved on average. In total, 68 patients were included in the analysis, of which 494 oocytes were measured and 758 oocytes were not measured (see Table 1).

**Table 1:** Study overview. A split cohort study was conducted at two IVF clinics.

	Patients	Measured	Control	Culture	Age	MII
**Shenzhen Army Hospital**	46	279	480	day 3	29.85 (21–39)	16.74
**Taiwan IVF Group Center**	22	215	278	day 5/6	34.36 (28–41)	22.45

After obtaining IRB approval from recruitment sites’ local IRB (Ton-Yen hospital JIRB: 18-001-A-1, Shenzhen Army Hospital IRB: 13751003667), all patients with greater than sixteen metaphase II oocytes (mature oocyte), and planning IVF with intracytoplasmic sperm injection (ICSI) using ejaculated sperm were recruited for participation from December 2016 to April 2021. This split cohort study was designed so that at most one half of study participants’ oocytes would be measured (experimental group) prior to fertilization with ICSI, with the remaining oocytes being fertilized without measurement (control group).

Patient stimulation was according to their treating physician, and no adjustments to their treatment plan were made due to their participation in the study. Oocyte retrieval was performed 36 hours after hCG administration, cumulus cells were removed 38 hours following hCG administration, and measurements were performed 40–42 hours following hCG administration, immediately prior to ICSI.

All oocytes underwent ICSI within 40–42 hours following hCG administration. Measured oocytes were individually cultured following injection, while the unmeasured cohort underwent group culture. Culture was performed in single step LifeGlobal media under low oxygen (5%) conditions. Fertilization, day 3 hatching, day 3 morphology assessment, and day 5/6 blastocyst assessments were performed as routinely done in the participating study sites.

Due to regulatory differences, the Shenzhen Army Hospital primarily cultured to day 3, while the Taiwan IVF Group Center primarily cultured to blastocyst. Subsequently, oocytes of participants from both clinics were used to assess fertilization and day 3 development, while oocytes of Taiwan IVF Group Center participants were primarily used to assess blastocyst development (see S1 Table). Successful fertilization denotes the formation of an embryo from a sperm and an egg. A good day 3 embryo was any embryo with grade A (7–8 cells on day 3). A blastocyst formation was any blastocyst with a Gardner grade of 3BC or better by day 5/6, while a usable blastocyst is chosen for transfer or vitrification as assessed by experienced embryologists. The parameters considered in the evaluation of oocyte anomality included oocyte cytoplasmic dysmorphisms, and extracytoplasmic dysmorphisms, while the optimal oocyte morphology is characterized by a spherical structure enclosed by a uniform zona pellucida, a uniform translucent cytoplasm, and a size-appropriate polar body [16].

### Determining the biomechanical properties of oocytes

**Fig 2:**
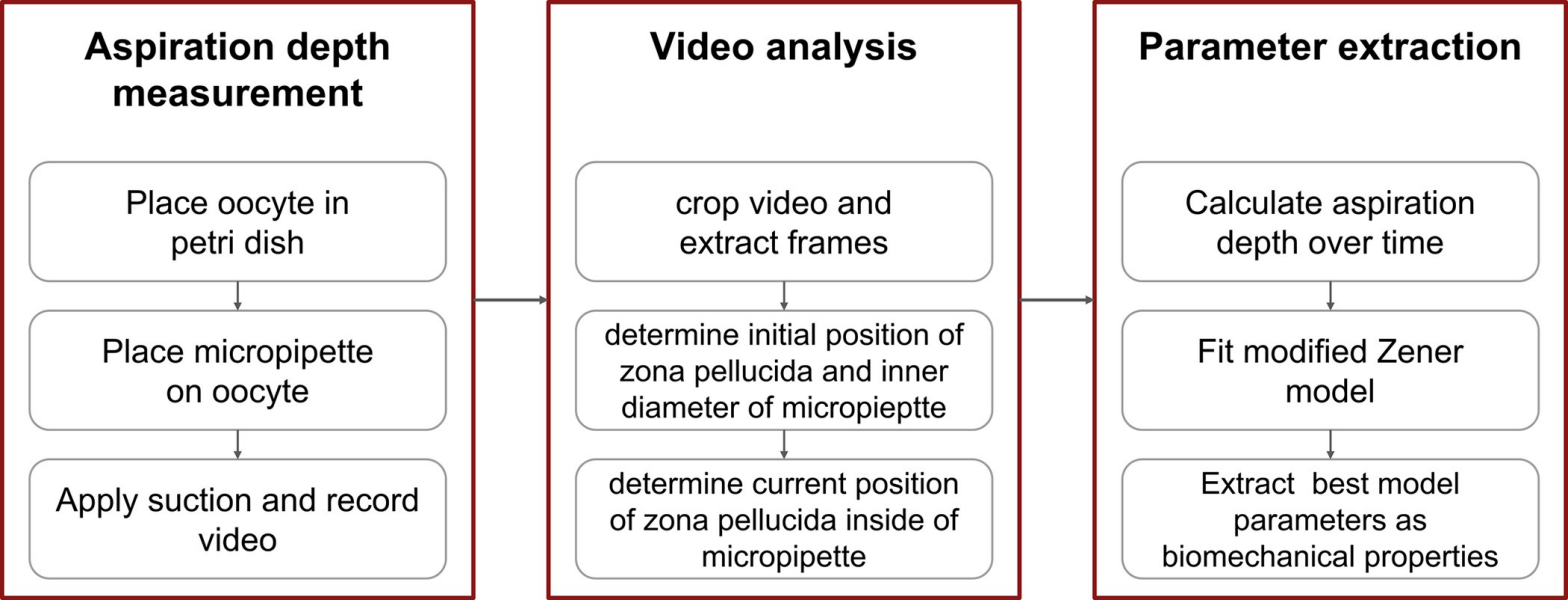
Workflow for determining the biomechanical properties of the oocytes. The workflow is divided into aspiration depth measurement, video analysis, and parameter extraction.

The measurement of biomechanical properties of retrieved oocytes from stimulated ovarian provides adjunctive information to aid the selection of oocytes with higher likelihood of developing into usable blastocysts. The biomechanical properties of the oocytes were extracted in three steps as shown in Fig 2 using similar micropipette aspiration device to the one used by Yanez et. al [15].

First, an aspiration depth measurement was performed and the displacement of an oocyte inside a micropipette over time was recorded. Secondly, the recorded video was analyzed and the aspiration depth over time was calculated. Finally, a modified Zener model (see sector of Parameter extraction) was fitted to the experimental data, providing biomechanical properties of the model as the representation of the biomechanical properties of the oocytes.

#### Aspiration depth measurement

**Fig 3:**
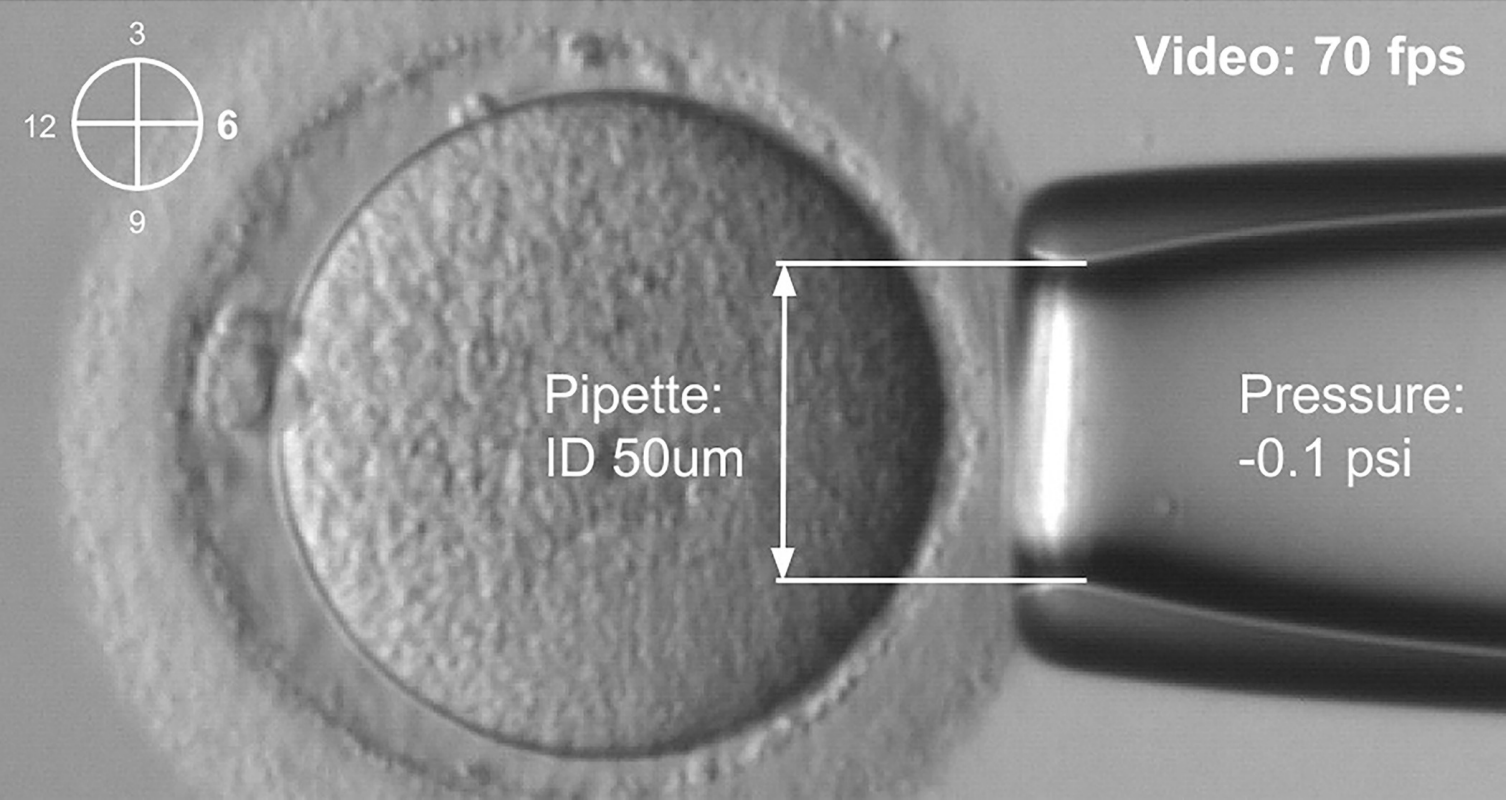
Aspiration depth measurement procedure. A small suction is applied to the oocyte through a micropipette. The procedure is recorded with a video camera at 70 frames per second.

Oocytes, placed in individual drops of modified human tubal fluid media, were measured, and individually tracked for outcomes. A micropipette was placed on the zona pellucida directly opposite to the location of the polar body (see Fig 3) to standardize the location of measurement in an area, a procedure performed similarly to the previous study [15]. Given the larger size of a human oocyte (~110 μm) as compared to that of a mouse embryo (~80 μm), 50-μm inner diameter of micropipettes (custom-made by Origio) and an applied pressure (-0.1 p.s.i.), which was modified to be slightly larger than the 40-μm inner diameter of micropipettes in Yanez et. al [15] and the applied pressure of ~0.5 kPa (~0.073 p.s.i.) in Young’s modulus for mouse embryo [17], were used and applied. The pressure was applied through a micropipette for 2 seconds and the movement of the zona pellucida inside the micropipette was recorded at 70 frames per second with a CMOS camera (Thorlabs DCC1545M). The pressure was regulated back to the balance pressure after each measurement to release the oocyte from the micropipette tip. The operator could easily navigate through the measurement steps displayed on the computer screen or pressure log file. This included monitoring pressure values both before and during measurements, allowing them to assess the success of each measurement.

#### Video analysis

**Fig 4:**
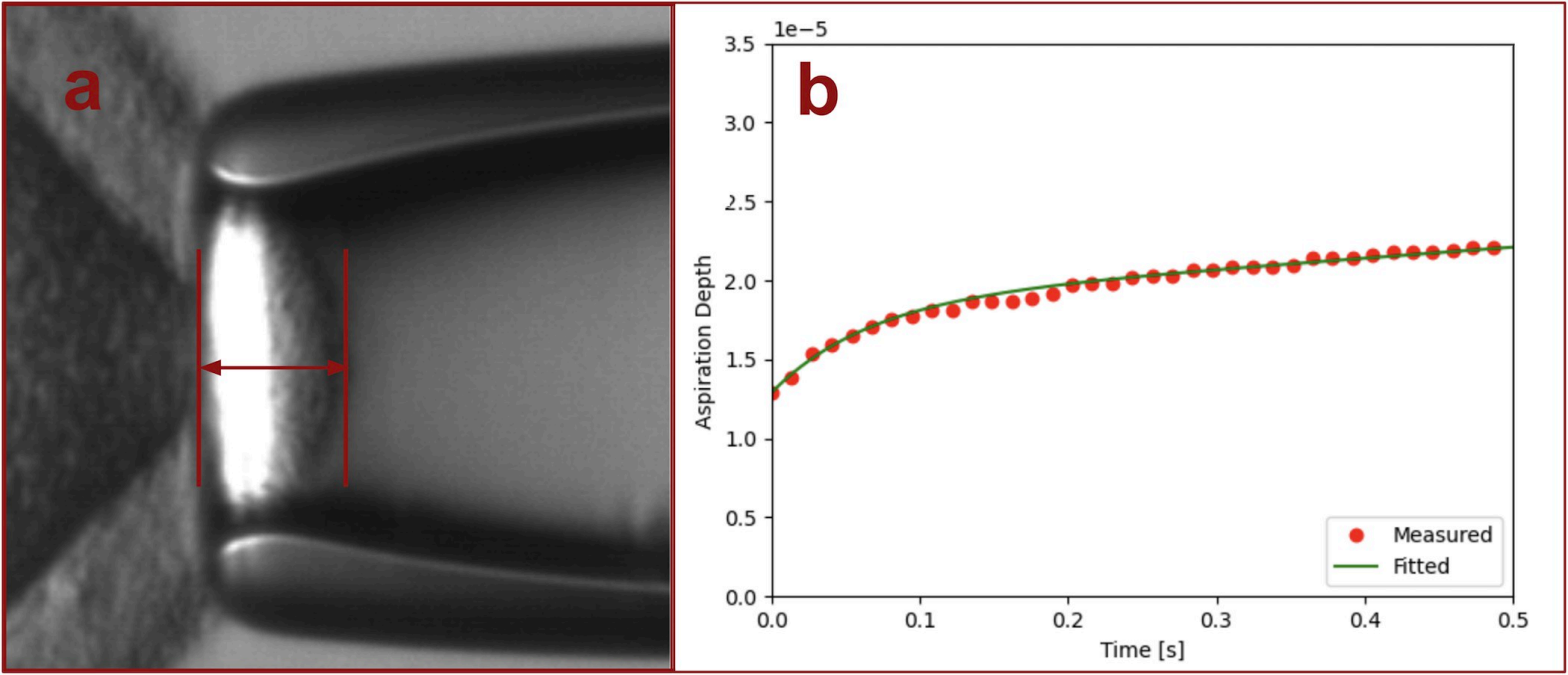
Aspiration depth video analysis and model fitting. **(**a) The photo shows an oocyte that had been partially pulled into the micropipette. The aspiration depth was measured between the position of the zona pellucida and the tip of the micropipette. (b) The picture shows an example of the measured aspiration depth and the modeled aspiration depth of an oocyte.

Before analyzing the aspiration depth, each recorded video was clipped to a length of 0.5 seconds starting with one frame before the zona pellucida started to move. The clipped video was further extracted each of frames. Additionally, the thickness of the zona pellucida, the inner diameter of the micropipette and the aspiration depth of zona pellucida inside the micropipette were labeled manually for further usage of analysis. The thickness of the zona pellucida and the inner diameter of the micropipette were determined in the first frame. The subsequent frames were used to determine the locations of the zona pellucida inside of the micropipette during the aspiration. The aspiration depth was calculated relative to the micropipette tip (see Fig 4A). The resulting aspiration depth in pixels was then converted to micrometers with a conversion factor that is the ratio of an object of known size and its pixels under the microscope.

**Parameter extraction (**mechanical properties**)**: Because oocytes exhibit elasticity as well as viscous properties, we applied a modified Zener model (Modified Linear Elastic Solid Model) with two springs and two dashpots to represent the mechanical (viscoelastic) properties of oocytes as it was aspirated into the micropipette [15]. As such, the model can take into account both kinds of behavior associated with the oocyte and the equation for aspiration depth over time *d*(*t*) can be represented as below:

d(t)=F0k1*(1−k0k0−k1e−tτ)−t*F0η1

$$where\;\tau =\frac{\eta_{0}*{(k}_{0}+k_{1})}{k_{0}*k_{1}}$$

The aspiration depth over time *d*(*t*) is given by the elongation of the modified Zener model when applying the applied force *F*_0_ that is produced by the pressure inside the micropipette. The equation of the applied force *F*_0_ is calculated as below:

$$F_{0}=pressure*area=pressure*p.s.i*{\left(\frac{d}{2}*\mu \right)}^{2}*\pi$$

where the unit of *pressure* is psi generated by pressure device over time, where *p.s.i* is a ratio of Pa to p.s.i, *d* is the inner diameter of micropipette, *μ* is a ratio of m to μm, and *π* is a ratio of a circle’s circumference to its diameter.

The modified Zener model is fitted to the experimental data by minimizing the sum of squared errors (SSE) between the measured aspiration depth and the modeled aspiration depth using the Broyden-Fletcher-Goldfarb-Shanno (BFGS) algorithm [18] (see Fig 4B). The resulting mechanical parameters (η_0_, η_1_, k_0_, k_1_, and τ) for the modified Zener model that best fitted the experimental data can be described as biomechanical (or viscoelastic) properties of the oocytes in this study. The parameters η_0_ and η_1_ can be represented as liquid-like behavior (viscous properties) of the oocyte, while the parameters k_0_ and k_1_ can be represented as solid-like behavior (elastic properties) of the oocyte, which describes the “instant elongation” when the forces are applied on the oocyte. The parameter τ can be represented as how fast (e.g., speed) the oocyte deforms after the initial instant elongation.

### Data analysis

#### Safety analysis

Embryo development outcomes from two clinics were analyzed to determine if the aspiration depth measurements (experimental group) affect embryo development when compared to the unmeasured group (control group). We used a chi-square test to determine if the fertilization (normal fertilization vs fertilization failure), good day 3 embryo (Grad A vs Non-Grade A) and blastocyst formation (any blastocyst vs no blastocyst) rates were statistically different between the experimental and the control groups.

#### Predictive modeling

**Fig 5:**
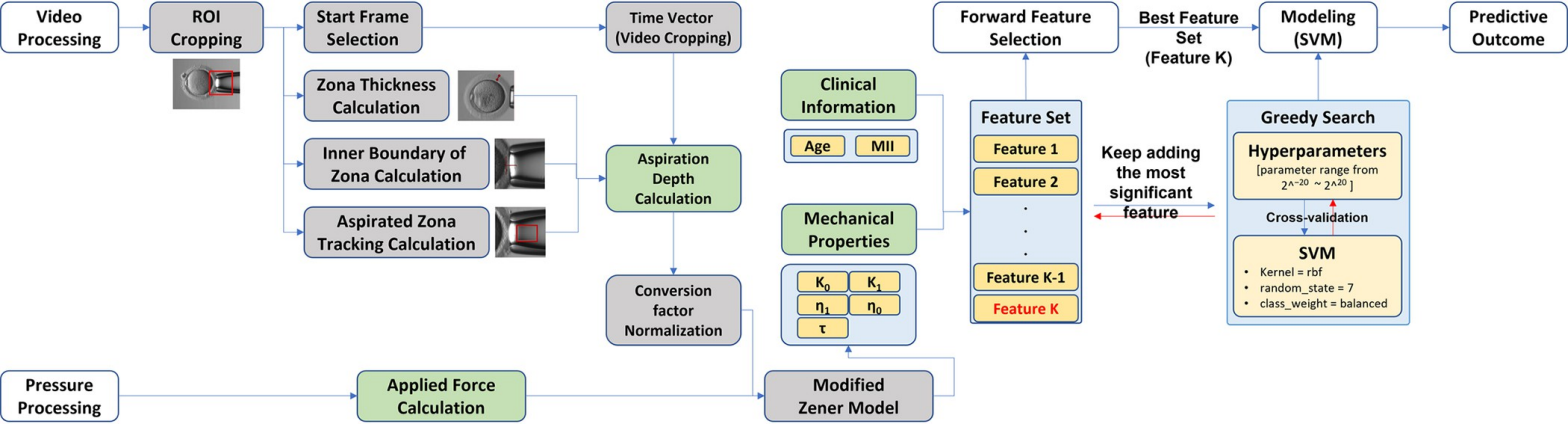
Modeling diagram. In the **Video processing, ROI (region of interest) Cropping** zooms in on the video to measure the inner boundary of zona pellucida, inner diameter of micropipette, and aspiration depth inside the micropipette. **Start Frame Selection** starts videos from a frame before the first moved frame. **Zona Thickness Calculation** calculates the thickness of zona pellucida as an initial aspiration depth. **Inner Boundary of Zona Calculation** determines the position of the inner zona pellucida near the micropipette tip. **Aspirated Zona Tracking Calculation** detects the position of zona pellucida inside of micropipette each frame over time. **Time Vector** keeps moved frames in the video and shrinks the video length to reduce the analysis time. **Aspiration Depth Calculation** calculates the aspiration depth of oocyte each frame over time. **Conversion Factor Normalization** standardizes the differences in video resolution by normalizing the aspiration depth with the inner diameter size of micropipette. In the **Pressure processing, Applied Force Calculation** measures the pressure force applied on the oocyte when aspirated into micropipette. **The Modified Zener Model** finds the mechanical parameters of viscoelastic behavior of oocyte as features of biomechanical properties for the predictive classifier. **Forward Feature selection** determines the best combination of features between **clinical information** and **mechanical properties** for model training. **Modeling** uses greedy search to find the best parameters for building a SVM classifier during cross-validation procedure. **Predictive outcome** provides a result indicating whether the blastocyst is usable or unusable.

Based on the measurement data from the experimental group at the Taiwan IVF Group Center, a total of 215 measured oocytes were initially included. However, 6 measured oocytes were excluded due to poor video quality, resulting in a final sample size of 209 measured oocytes used for model building. We further created two datasets for building a usable blastocyst classifier, where the first dataset contained all data (209 measured oocytes), and the second dataset included only the fertilized oocytes (160 measured oocytes) with the intention of retaining the factors necessary for normal fertilization. The estimated mechanical parameters (η_0_, η_1_, k_0_, k_1_ and τ) and the two maternal factors (patient age, and the number of retrieved mature oocytes (MII)) were extracted as the features for building the predictive classifier (see Fig 5). To normalize skewed data and scale features with large ranges, all features were log2 transformed and then standardized by removing the mean and scaling to unit variance. We trained a predictive classifier using Support Vector Machine [19] to predict the outcomes of the usable blastocyst development. The dataset was split by patients into a training set (~70% of the data) and a test set (~30% of the data). We trained the classifiers by maximizing the area under the curve (AUC) of a receiver operating characteristic (ROC) using 10-fold cross validation. Forward feature selection [20] was used to extract the most useful features for classification.

### Ethics statement

Ethical approval was obtained from the Joint Institutional Review Boards of Ton-Yen Hospital (JIRB: 18-001-A-1, recruitment period: April 1, 2018, to April 23, 2021), and Shenzhen Army Hospital Ethics Committee (IRB: 13751003667, recruitment period: December 2016 to December 2018). All patients provided informed written consent for their oocytes to be used in this study and gave permission for researchers to access medical records to obtain their reproductive history and IVF outcomes.

## Results

### Embryo development is not negatively affected by the aspiration measurement procedure

**Fig 6:**
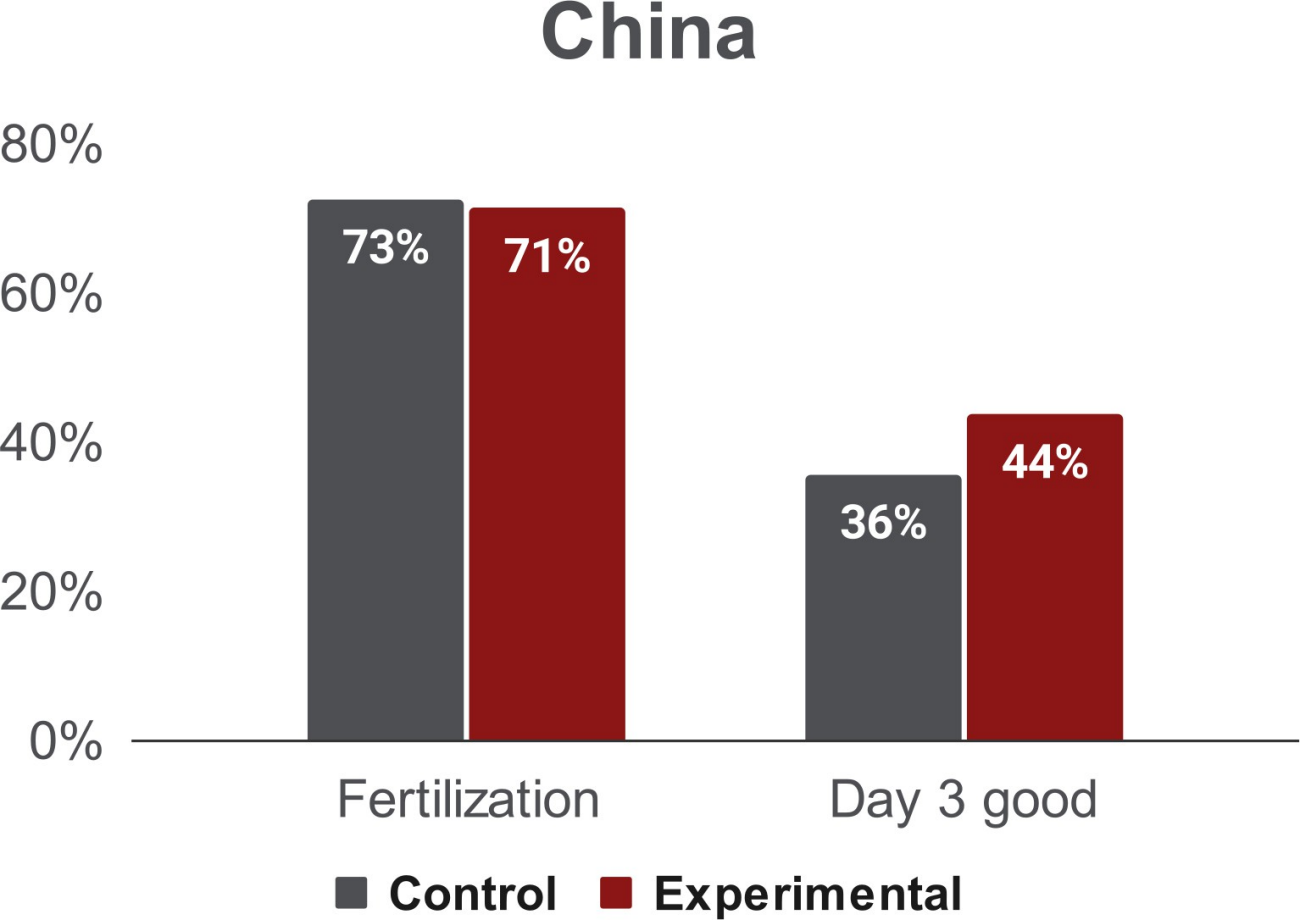
Outcomes from the Shenzhen Army Hospital. Fertilization rates and day 3 good embryo rates of the control and the experimental groups. The fertilization rate was not significantly different between the two groups (p = 0.7282), while the day 3 good embryo rates were not negatively affected by the aspiration measurement in the experimental group (p<0.01).

**Fig 7:**
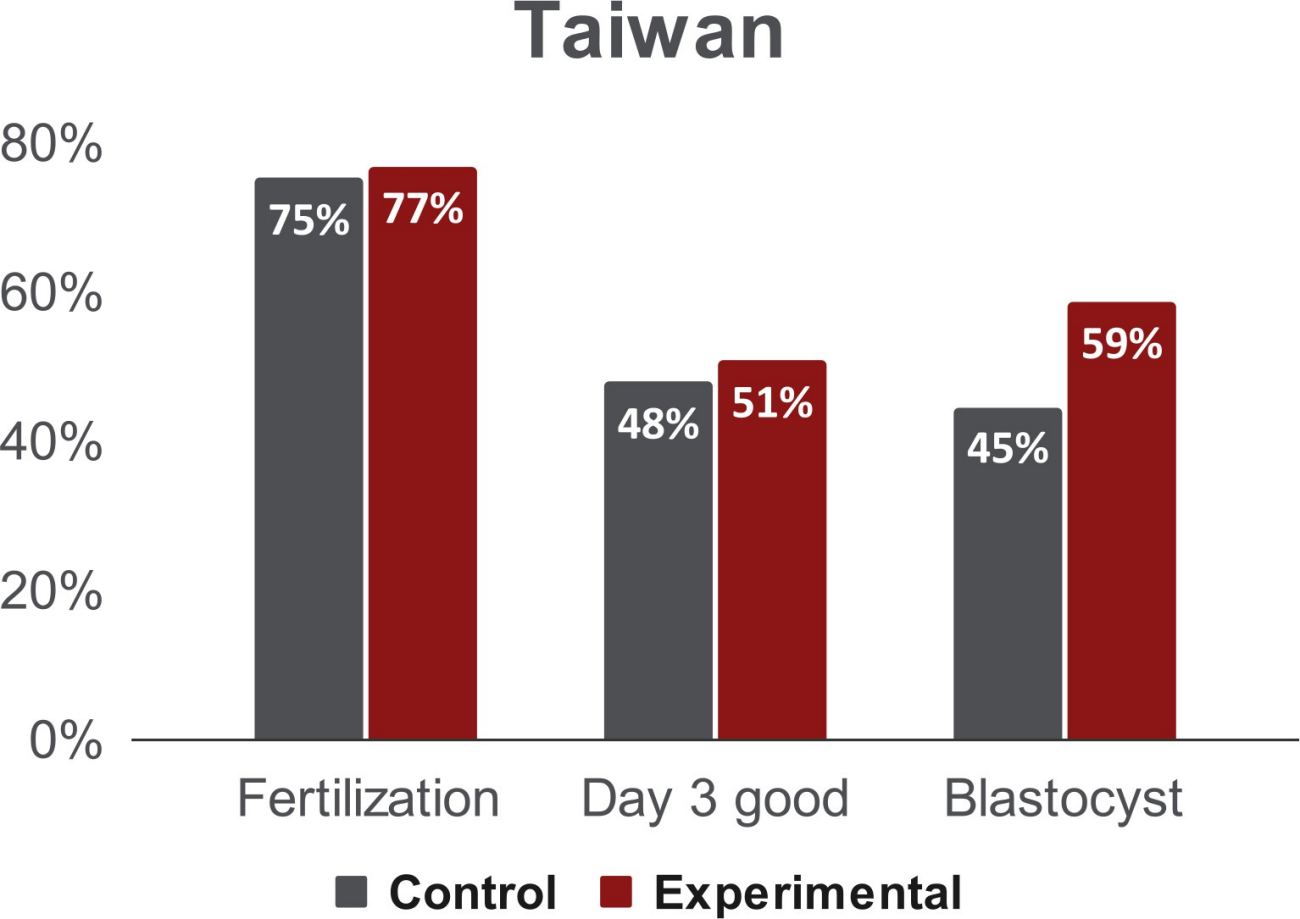
Outcomes from the Taiwan IVF Group Center. Fertilization rate, day 3 good embryo rates and blastocyst formation rates of the control and the experimental group. The fertilization and day 3 good embryo rates were not significantly different between the two groups (p = 0.69 experimental and p = 0.63 control), the blastocyst formation rates were not negatively affected by the aspiration measurement in the experimental group (p<0.01).

Following the aspiration procedure, we investigated whether aspiration depth measurements (experimental group) affected embryo development, as compared to the control group (no measurements). We examined fertilization rates, good day 3 embryo rates, and blastocyst formation rates (see Fig 1). At the Shenzhen Army Hospital, fertilization rates were not significantly different between the two groups (p = .7282). However, the rate of good day 3 embryos was significantly higher in the experimental group (see Fig 6; p < .01). At the Taiwan IVF Group Center, fertilization rates and day 3 good embryo rates were not significantly different (p = .69 and p = .63, respectively). The blastocyst formation rate was significantly higher in the experimental group (see Fig 7; p < .01). These results suggest that oocytes are not negatively affected by the aspiration measurement procedure.

### Biomechanical properties of oocytes correlated with the development potential of preimplantation embryos

Currently, there are no standard criteria or grading system for selecting oocytes with a higher likelihood of developing into usable blastocysts. This study investigated the link between biomechanical properties of oocytes and blastocyst development. First, 146 (~70% of the training set) out of 209 measured oocytes from the Taiwan IVF Group Center were used to train a predictive classifier that would predict the formation of usable blastocysts on day 5/6. Next, 63 (~30% of the test set) out of 209 measured oocytes were used to validate the predictive classifier with four optimal features (τ, k0, k1, and η0) as the biomechanical properties, achieving an accuracy of 71% (see Table 2).

**Table 2:** Prediction results of the usable blastocysts predictive classifier with biomechanical properties compared to maternal factors and morphological information by four embryologists.

	Biomechanical properties ^b^	Maternal factors ^c^	Mixed features ^d^	Emb1 ^e^	Emb2 ^e^	Emb3 ^e^	Emb4 ^e^
**ACC** ^**a**^ **[%]**	71	57	52	38	42	50	44
**PPV** ^**a**^ **[%]**	68	55	51	40	39	47	33
**NPV** ^**a**^ **[%]**	77	61	75	48	43	48	47
**SEN** ^**a**^ **[%]**	81	71	97	19	35	58	13
**SPE** ^**a**^ **[%]**	63	44	9	72	47	38	75

^**a**^ Accuracy (ACC), Positive Predictive Value (PPV), Negative Predictive Value (NPV), Sensitivity (SEN), Specificity (SPE).

^**b**^ Predictive classifier was trained using 209 measured oocytes, which included four optimal biomechanical features.

^**c**^ Predictive classifier was trained using 209 measured oocytes, which included maternal factors of patient age and number of MII.

^**d**^ Predictive classifier was trained using 209 measured oocytes, which included four optimal biomechanical features, patient age, and number of MII.

^**e**^ Prediction results predicted by experienced embryologists.

Four embryologists were separately asked to predict usable blastocysts based on the morphological information of oocytes in the test dataset. The resulting accuracies were 38%, 42%, 50%, and 44% (see Table 2). We also measured the multi-rater reliability between four experienced embryologists and achieved a poor agreement (Fleiss’ Kappa score: 0.08 when assessing the morphology of oocytes), indicating that embryologists may have subjective opinions on the selection of oocyte.

Further analysis showed that the predictive classifier was more accurate than maternal factors (patient age and number of mature oocytes (MII)) (accuracy of 57%) in comparison. Unfortunately, incorporating four biomechanical properties with maternal factors led to a decrease in accuracy, resulting in a 52% accuracy rate. The results above indicate that biomechanical properties demonstrate more accuracy in predicting usable blastocyst formation (see Table 2).

## Discussion

This study demonstrated that measurements of biomechanical or viscoelastic properties of oocytes can be performed without negatively affecting the embryo development through micropipette aspiration. Further analysis is needed to confirm that the combination of measurement with micropipette aspiration and freezing/thawing does not result in more damage than either individually, as cryopreservation and thawing may affect the spindle [21]. While better day 3 good embryo development was observed at the Shenzhen Army Hospital and higher blastocyst formation rates were observed at the Taiwan IVF Group Center in the measured cohort, a more stringent and well-designed study will be required for further validation. Other factors, like the fact that unmeasured oocytes were cultured together whereas measured oocytes were grown independently, could have affected development.

Biomechanical properties were used in this study to predict usable blastocyst formation on day 5/6. The predictive value of these parameters was more significant in the cohort of oocytes that fertilized normally than the overall cohort of measured oocytes, suggesting that biomechanical properties are poorly predictive of normal fertilization. This is intuitive, given the other variables are required for normal fertilization after ICSI including sperm quality and injection technique. A predictive classifier using biomechanical properties yielded the most predictive results in the cohort of normally fertilized oocytes. Although limited, the accuracy exceeded the subjective analysis performed by embryologists, which had a poor reliability of agreement (or reproducibility) between observers (Fleiss’ Kappa score: 0.08) and low accuracy. These findings show the need for more accurate assessments of oocyte reproductive potential and that the use of biomechanical properties of oocytes as a predictor could be helpful in improving embryo selection.

In the previous study [15], the k_0_, k_1_ and η_1_ parameters were also used as biomechanical properties to evaluate the human embryo developmental potential using the zygote. In this study, τ, k_0_, k_1_, and η_0_ parameters were selected as optimal features. For a better understanding of the biomechanical properties of oocytes, we also compared each interquartile of each property of oocyte that fertilized normally. The k_1_ parameter resulted in the significantly highest accuracy, indicating its significance and importance among the various biomechanical properties (see S2 Table). The τ parameter is the harmonic mean of the k_0_, and k_1_ and can be adjusted for the intensity by weighting η_0_ parameter. It thus symmetrically represents 3 parameters of the k_0_, k_1_ and η_0_ in one metric, indicating the τ parameter may be able to exhibit the elasticity as well as viscous properties of oocytes.

Time-lapse has been used widely to predict blastocyst formation and quality based on the analysis of embryo development from day 1 to 3 [22]. While the technology has reduced the time required to observe embryo development, its efficacy in predicting blastocyst quality for transfer remains inconclusive [23]. In this study, the biomechanical properties of oocytes were found to be correlated with usable blastocyst formation, which could potentially be used as an indicator of blastocyst quality prior to transfer.

Several studies indicated that alterations in oocytes stiffness may affect fertilization, embryo viability, genetic abnormality, and pregnancy [11–14], suggesting that viscoelasticity could serve as an indicator in the aspiration measurement prior to ICSI or classical IVF procedure. However, a more stringent and well-designed study will be required to establish this as for proof-of-concept. Additionally, the possibility of chromosomal abnormality from the sperm will need to be considered, as well as the accuracy of PGT-A.

Several studies indicated that sperm quality or other sperm factors may impact the fertilization [24,25]. However, the biomechanical properties of oocytes may not predict fertilization failure caused by poor sperm quality. To test this assumption, out of the 160 measured oocytes that fertilized successfully, 112 (~70% of the training set) were used to develop a classifier that could predict the formation of usable blastocysts on day 5/6. Additionally, 48 (~30% of the test set) were used to validate the accuracy and effectiveness of the predictive classifier. The results showed an accuracy of 81% on the test dataset (see S3 Table), which supported our assumption that factors such as sperm quality are critical for normal fertilization. In conclusion, using dynamic deformation analysis of oocytes as a predictor could be helpful in improving blastocyst selection.

## Conclusion

A predictive classifier was able to use biomechanical properties to predict the developmental potential of mature oocytes with greater accuracy than experienced embryologists. The measurement procedure did not negatively affect embryo culture outcomes. While further analysis is necessary, this study shows the potential of using biomechanical properties of oocytes to predict developmental outcomes. However, this study’s application is limited to a general infertility or fertility preservation population, and its findings should be reproduced in more diverse, poorer prognosis patients before being applied more broadly.

## Supporting information

S1 Appendix Information of oocyte development. (XLSX)

S1 Table Oocyte development information. (DOCX)

S2 Table Interquartile comparison of each biomechanical property of oocyte that fertilized normally. (DOCX)

S3 Table Results of usable blastocysts prediction by the predictive classifier. (DOCX)
