# Supplementary material for: Development and evaluation of a usable blastocyst predictive model using the biomechanical properties of human oocytes
Source: PLoS One. 2024 May 2;19(5):e0299602. doi: 10.1371/journal.pone.0299602 (PMC11065297; doi:10.1371/journal.pone.0299602)
Supplement: S1 Table — (DOCX) [file pone.0299602.s002.docx]

**S1 Table. Oocyte development information.**

|  | | **Fertilization** | | **Good day 3 embryo ^a^** | | **Any blastocyst ^b^** | | **Usable Blastocyst ^c^** | |
| --- | --- | --- | --- | --- | --- | --- | --- | --- | --- |
|  | | **Yes** | **No** | **Yes** | **No** | **Yes** | **No** | **Yes** | **No** |
| **Shenzhen Army Hospital** | **Control** | 348 | 132 | 171 | 309 | - **^d^** | - | - | - |
|  | **Experimental** | 199 | 80 | 122 | 157 | - | - | - | - |
| **Taiwan IVF Group Center** | **Control** | 209 | 69 | 133 | 145 | 124 | 154 | 100 | 178 |
|  | **Experimental** | 165 | 50 | 109 | 106 | 126 **^e^** | 89 | 107 | 108 |

**^a^** A good day 3 embryo was any embryo with grade A (7-8 cells on day 3).

**^b^** A blastocyst formation was any blastocyst with a Gardner grade of 3BC or better by day 5/6.

**^c^** A usable blastocyst is chosen for transfer or vitrification as assessed by experienced embryologists.

**^d^** Due to regulatory differences, the Shenzhen Army Hospital primarily cultured to day 3 embryo development.

**^e^** Embryos with a low grade have a chance of developing into a blastocyst.
