# Supplementary material for: Development and evaluation of a usable blastocyst predictive model using the biomechanical properties of human oocytes
Source: PLoS One. 2024 May 2;19(5):e0299602. doi: 10.1371/journal.pone.0299602 (PMC11065297; doi:10.1371/journal.pone.0299602)
Supplement: S2 Table — (DOCX) [file pone.0299602.s003.docx]

**S2 Table. Interquartile comparison of each biomechanical property of oocyte that fertilized normally.**

|  | **Q1 ^b^** | **Q2 ^b^** | **Q3 ^b^** | **Q4 ^b^** | **P-value ^c^** |
| --- | --- | --- | --- | --- | --- |
| **η_0_ ^a^** | 75% **^d^** | 68% | 58% | 55% | 0.218 |
| **η_1_ ^a^** | 65% | 70% | 63% | 58% | 0.704 |
| **k_0_ ^a^** | 70% | 68% | 63% | 55% | 0.518 |
| **k_1_ ^a^** | 75% | 78% | 58% | 45% | **0.007** |
| **τ ^a^** | 73% | 68% | 58% | 58% | 0.404 |

**^a^** Biomechanical property of oocyte.

**^b^** Interquartile of each biomechanical property.

**^c^** P-values were calculated by chi-square test.

**^d^** Usable blastocyte Rate.
