# Supplementary material for: Development and evaluation of a usable blastocyst predictive model using the biomechanical properties of human oocytes
Source: PLoS One. 2024 May 2;19(5):e0299602. doi: 10.1371/journal.pone.0299602 (PMC11065297; doi:10.1371/journal.pone.0299602)
Supplement: S3 Table — (DOCX) [file pone.0299602.s004.docx]

**S3 Table. Results of usable blastocysts prediction by the predictive classifier.**

|  | **Predictive classifier ^b^** |
| --- | --- |
| **ACC ^a^ [%]** | 81 |
| **PPV ^a^ [%]** | 82 |
| **NPV ^a^ [%]** | 79 |
| **SEN ^a^ [%]** | 90 |
| **SPE ^a^ [%]** | 65 |

**^a^** Accuracy (ACC), Positive Predictive Value (PPV), Negative Predictive Value (NPV), Sensitivity (SEN), Specificity (SPE).

**^b^** Predictive classifier was trained using 160 measured oocytes that fertilized successfully, which included four optimal biomechanical features.
